# Increasing screening for atrial fibrillation in general practice: the Atrial Fibrillation Self‐Screening, Management And guideline‐Recommended Therapy (AF Self‐SMART) study

**DOI:** 10.5694/mja2.51803

**Published:** 2022-12-09

**Authors:** Katrina Giskes, Nicole Lowres, Jessica Orchard, JiaLin Li, Kirsty McKenzie, Charlotte Mary Hespe, Ben Freedman

**Affiliations:** ^1^ Heart Research Institute, the University of Sydney Sydney NSW; ^2^ The University of Notre Dame Australia Sydney NSW; ^3^ Charles Perkins Centre, the University of Sydney Sydney NSW; ^4^ Centenary Institute, the University of Sydney Sydney NSW

**Keywords:** Atrial fibrillation, General practice, Technology

## Abstract

**Objective:**

To assess whether atrial fibrillation (AF) self‐screening stations in general practice waiting rooms improve AF screening, diagnosis, and stroke risk management.

**Design, setting:**

Intervention study (planned duration: twelve weeks) in six New South Wales general practices (two in rural locations, four in greater metropolitan Sydney), undertaken during 28 August 2020 – 5 August 2021.

**Participants:**

People aged 65 years or more who had not previously been diagnosed with AF, and had appointments for face‐to‐face GP consultations. People with valvular AF were excluded.

**Intervention:**

AF self‐screening station and software, integrated with practice electronic medical record programs, that identified and invited participation by eligible patients, and exported single‐lead electrocardiograms and automated evaluations to patients’ medical records.

**Main outcome measures:**

Screening rate; incidence of newly diagnosed AF during intervention and pre‐intervention periods; prescribing of guideline‐recommended anticoagulant medications.

**Results:**

Across the six participating practices, 2835 of 7849 eligible patients (36.1%) had face‐to‐face GP appointments during the intervention period, of whom 1127 completed AF self‐screening (39.8%; range by practice: 12–74%). AF was diagnosed in 49 screened patients (4.3%), 44 of whom (90%) had CHA_2_DS_2_‐VA scores of 2 or more (high stroke risk). The incidence of newly diagnosed AF during the pre‐intervention period was 11 cases per 1000 eligible patients; during the intervention period, it was 22 per 1000 eligible patients (screen‐detected: 17 per 1000 eligible patients; otherwise detected: 4.6 per 1000 eligible patients). Prescribing of oral anticoagulation therapy for people newly diagnosed with AF and high stroke risk was similar during the pre‐intervention (20 of 24, 83%) and intervention periods (46 of 54, 85%).

**Conclusions:**

AF self‐screening in general practice waiting rooms is a feasible approach to increasing AF screening and diagnosis rates by reducing time barriers to screening by GPs. AF self‐screening could reduce the number of AF‐related strokes.

**Trial registration:**

Australian New Zealand Clinical Trials Registry ACTRN12620000233921 (prospective).

**The known:** Atrial fibrillation (AF) screening is recommended for people aged 65 years or more to avert preventable stroke, but general practitioners screen only about 11% of eligible patients, often because of time constraints.

**The new:** Our self‐screening stations in general practice waiting rooms, coupled with custom software that automatically identified eligible patients in electronic practice records and transferred their single‐lead electrocardiograms and evaluations to their medical records, achieved a screening rate almost four times as high as that found by a survey of standard practice, and doubled the AF diagnosis rate.

**The implications:** Our fully integrated approach to AF self‐screening could overcome time problems for GPs and increase screening rates and reduce the number of AF‐related strokes.

About 10% of ischaemic strokes are attributed to newly diagnosed atrial fibrillation (AF).^1^ Two in three people with AF have no or atypical symptoms.^2^ Early detection and initiation of anticoagulation treatment for people at high risk can reduce the number of avoidable strokes.^1^ As opportunistic AF screening is recommended by Australian^3^ and European guidelines^4^ for people aged 65 years or more, a feasible screening strategy in a setting with an efficient diagnosis and management pathway is required.

General practice is an ideal setting for AF screening. Each year, more than 90% of Australian adults consult general practitioners,^5^ but only 10–15% of GPs regularly screen patients for AF.^6^ We have reported that integrating prompts for screening with handheld electrocardiography devices into general practice software achieved a mean screening rate in metropolitan and rural general practices of 34% (range: 9–51%).^7^, ^8^ However, time constraints were the main barrier to AF screening for both GPs and practice nurses,^9^ as also reported by overseas investigators.^10^, ^11^

The aim of the AF Self‐SMART (Self‐Screening, Management And guideline‐Recommended Therapy) study was to design and set up AF self‐screening stations in general practice waiting rooms, and to determine whether the stations improved AF screening, diagnosis, and stroke risk management in general practice.

## Methods

AF Self‐SMART was a cross‐sectional implementation study in six New South Wales general practices during 28 August 2020 to 5 August 2021. The study was registered with the Australian New Zealand Clinical Trials Registry on 25 February 2020 (ACTRN12620000233921), and a detailed study protocol has been published elsewhere.^12^

### Self‐screening station and software

A purpose‐built patient self‐screening station was installed in the waiting rooms of each participating general practice (Box 1). The station included an iPad with KardiaStation software (version 2.0.0; www.kardia.com) and a Kardia electrocardiography device (model AC‐009) for recording a 30‐second lead‐1 electrocardiogram (ECG). The KardiaPro algorithm classified each ECG as “normal”, “possible AF”, or “unclassified” (95% sensitivity and 99% specificity for detecting AF in a clinical setting).^13^ Custom software integrated the screening station into the practice electronic medical records software (Best Practice).^12^ The self‐screening station and software were refined during three months of pilot testing in one practice, and the screening station, patient instructions, and screening protocol were iteratively adjusted in each practice throughout the study (Supporting Information, supplementary methods).

Box 1 **Atrial fibrillation self‐screening station**

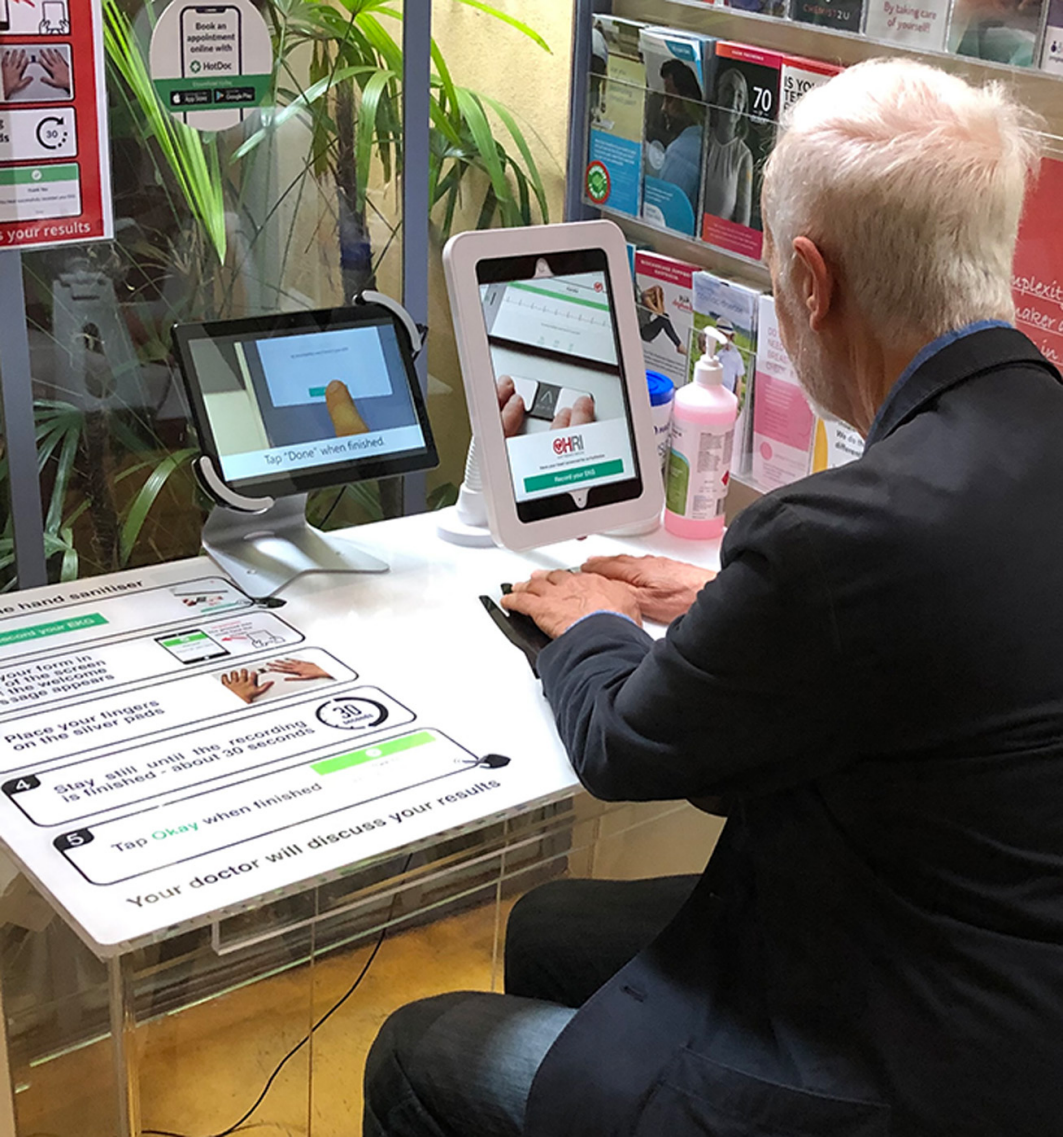

### Eligibility and consent

We recruited a convenience sample of practices in metropolitan and rural NSW from the research networks of the investigators and their colleagues (ie, general practices that have indicated interest in or have participated in research over the past ten years). We invited practices that had at least four fulltime GPs, used Best Practice medical records software, and had wireless internet networks. Each practice provided written consent for participation.

Eligible patients were aged 65 years or older, had not previously been diagnosed with AF, had face‐to‐face GP appointments on the day of self‐screening, and had not already undertaken AF self‐screening. Patients were provided with study information sheets, and their consent to participation was assumed if they completed AF self‐screening. We aimed to recruit five to eight general practices and a minimum of 1500 eligible patients to undertake AF self‐screening.

### Self‐screening procedure

The AF Self‐SMART software, integrated with the practice appointment diary, identified patients eligible for participation and sent them text messages about AF self‐screening. On arrival at the clinic for face‐to‐face consultations, reception staff provided each eligible patient with a printed quick response (QR) code and directed them to the self‐screening station. After scanning their QR code to register their personal details and applying hand sanitiser, patients followed iPad prompts to record a 30‐second ECG. The ECG and its algorithm‐based evaluation were instantly transmitted to the patient's investigations inbox (as for pathology reports); the treating GP could view the 30‐second ECG rhythm strip and its evaluation and discuss it with the patient during their consultation. Treating GPs had been trained in reviewing all traces not categorised as normal, and communicated the results to patients with evidence‐based guideline recommendations for AF management.^4^ GPs were also responsible for all decisions regarding further investigations and management of the patient.

### Data extraction

We extracted de‐identified data from electronic medical records for active patients, defined as those who had had at least three appointments with the practice during the preceding 24 months, including at least one during the past six months. For each practice, we extracted data for two time periods: the intervention period (planned duration: twelve weeks) and a pre‐intervention period of equal length preceding the first contact the research group had with the practice. Diagnosis and prescribing data were also extracted three months after self‐screening to allow for lags between AF diagnosis and the initiation of treatment. Patients with newly diagnosed valvular AF were not included in our analysis, as treatment recommendations would be different to those for people with non‐valvular AF. To minimise the misclassification of incident AF, a potential problem given the inherent limitations of electronic medical record data extracts, we checked the free‐text areas of medical records for AF diagnoses.

### Practice remuneration

Participating practices received a study establishment fee ($1000) for the time taken to instal and test the study software, train staff, and extract study data. Further stepwise remuneration was based on the number of patients who completed self‐screening (0–99 patients, $500; 100–199 patients, $1000; 200–299 patients, $1500; 300 or more patients, $1500); the maximum total remuneration was thus $5500 per clinic.

### Statistical analyses

Data on sex, comorbid conditions, and prescribing are reported as summary statistics. The incidence rate for AF in scope patients was estimated. The statistical significance of between‐group differences was assessed in analysis of variance (continuous variables) or Pearson χ^2^ tests (categorical variables). Analyses were undertaken in SPSS 26.0.0 (IBM); *P* < 0.05 was deemed statistically significant (two‐tailed).

### Ethics approval

The human research ethics committees of the University of Sydney (2019/382) and the University of Notre Dame Australia (019145S) approved the study.

## Results

Six NSW practices participated in the study, two in rural locations and four in greater metropolitan Sydney (Supporting Information, table).

### Intervention duration

The planned intervention duration was twelve weeks, but the actual duration varied between practices (range: 3–24 weeks; Supporting Information, table). One practice included data for the 12‐week pilot testing phase as well as the 12‐week intervention phase; two practices effectively ended data collection after three or six weeks because coronavirus 2019 (COVID‐19) restrictions reduced the overall number of face‐to‐face consultations, and COVID‐19 vaccinations were prioritised by the practices.

### Patient self‐screening

Across the six practices, 2835 of 7849 eligible patients (36.1%) had face‐to‐face GP appointments during the intervention period. The proportion of female patients was larger for patients with appointments than for those without appointments (58.1% *v* 53.9%); the proportions of patients who had vascular disease (peripheral arterial disease, myocardial infarction, aortic plaque; 15.1% *v* 11.9%), diabetes (15.8% *v* 10.5%), or hypertension (52.2% *v* 39.5%) were larger than for patients with appointments than for patients without appointments. The mean age of patients who had appointments was slightly higher (75.1 years; standard deviation [SD], 7.2 years) than for those who did not (74.1 years; SD, 7.3 years) (Box 2).

Box 2 **Characteristics of the 7849 eligible general practice patients from six practices, by face‐to‐face consultations during the study period (28 August 2020 – 5 August 2021) and self‐screening**

**Table:** 

	All eligible patients			Eligible patients with face‐to‐face appointments during study		
Characteristic	Face‐to face consultations	No face‐to face consultations	*P*	Self‐screening	No self‐screening	*P*
All patients	2835	5014		1127	1708	
Sex (women)	1646 (58.1%)	2701 (53.9%)	< 0.001	634 (56.3%)	1012 (59.3%)	0.28
Age (years), mean (SD)	75.1 (7.2)	74.1 (7.3)	0.008	74.9 (6.7)	75.2 (7.6)	0.29
Comorbid conditions						
Congestive heart failure	119 (4.2%)	190 (3.8%)	0.35	38 (3.4%)	81 (4.7%)	0.09
Stroke	197 (6.9%)	317 (6.3%)	0.26	72 (6.4%)	125 (7.3%)	0.38
Vascular disease	427 (15.1%)	597 (11.9%)	0.006	170 (15.1%)	257 (15.0%)	0.89
Diabetes	448 (15.8%)	526 (10.5%)	0.009	184 (16.3%)	264 (15.5%)	0.46
Hypertension	1481 (52.2%)	1982 (39.5%)	< 0.001	622 (55.2%)	859 (50.3%)	0.008

SD = standard deviation.

Of the 2835 eligible patients who had face‐to‐face consultations, 1127 completed AF self‐screening (39.8%; range by practice: 12–74%). The characteristics of those who completed screening and those who did not were similar, except that the proportion with hypertension was larger for those who completed self‐screening (55.2% *v* 50.3%) (Box 2).

### Newly diagnosed atrial fibrillation

Of the 1127 self‐screening ECGs, 51 were classified as indicating possible AF (4.5%), 848 as normal (75.2%), and 189 as unclassified (16.8%); 39 were unreadable or too short (3.5%) (Box 3). After GP consultation and further investigations, AF was diagnosed in 49 patients (4.3%), 44 of whom (90%) were deemed to be at high risk of stroke (CHA_2_DS_2_‐VA scores, calculated by the research team, of at least 2). Thirteen patients who did not undertake self‐screening (0.8%) were also diagnosed with AF during the study period, of whom ten had CHA_2_DS_2_‐VA scores of at least 2. The mean age of patients diagnosed with AF was higher for those in whom it was detected by self‐screening (79 years; SD, 8 years *v* 74 years; SD, 7 years) (Box 4).

Box 3 **Flow of eligible participants through the study**

**Figure:**
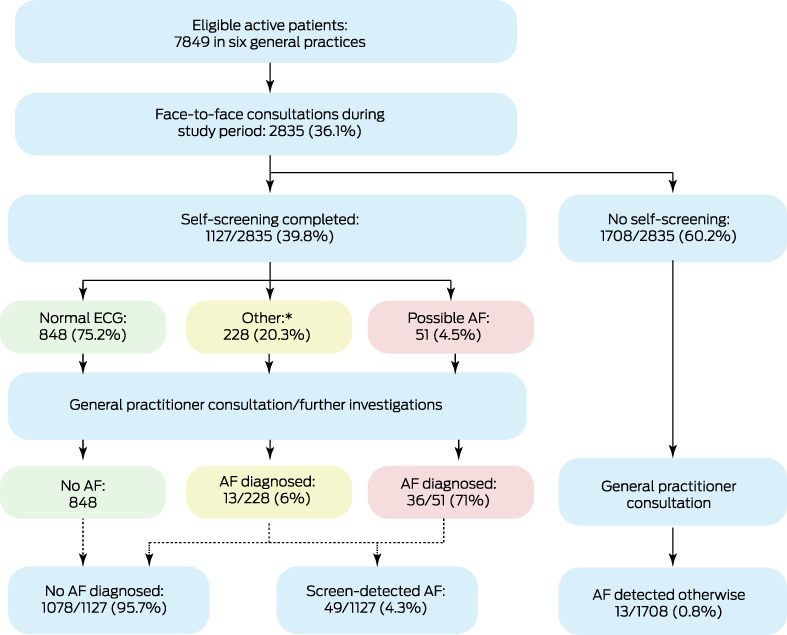
* Unclassified: 189 (16.8%); unreadable: 27 (2.4%); too short: 12 (1.1%).

Box 4 **Characteristics of patients diagnosed with atrial fibrillation (AF) before and during the self‐screening intervention, and stroke risk management for people identified as being at high risk**

**Table:** 

	Incident AF diagnosed prior to or during the intervention			Incident AF diagnosed during the intervention		
Characteristic	Prior to intervention	During intervention	*P*	Screen‐detected	Detected otherwise	*P*
All patients	27	62		49	13	
Sex (women)	12 (44%)	33 (53%)	0.45	22 (45%)	7 (54%)	0.57
Age (years), mean (SD)	78 (8)	76 (9)	0.42	79 (8)	74 (7)	0.018
Comorbid conditions						
Congestive heart failure	3 (11%)	5 (8%)	0.66	5 (10%)	0	0.23
Stroke	3 (11%)	4 (6%)	0.47	3 (6%)	1 (8%)	0.85
Vascular disease	7 (26%)	11 (18%)	0.40	11 (22%)	0	0.06
Diabetes	9 (33%)	8 (13%)	0.027	6 (12%)	2 (15%)	0.79
Hypertension	20 (74%)	43 (69%)	0.73	35 (71%)	8 (62%)	0.43
CHA_2_DS_2_‐VA score, mean (SD)	3 (1)	3 (1)	0.025	3 (1)	2 (1)	0.20
CHA_2_DS_2_‐VA score ≥ 2 (ie, high stroke risk)	24 (89%)	54 (87%)	0.53	44 (90%)	10 (77%)	0.06
Medical management of patients with CHA_2_DS_2_‐VA ≥ 2						0.20
Prescribed an oral anticoagulant*	20 (83%)	46 (85%)		37 (84%)	9 (90%)	
Prescribed antiplatelet medication only^†^	3 (13%)	5 (9%)		4 (9%)	1 (10%)	
No antithrombotic treatment prescribed	1 (4%)	3 (6%)	0.23	3 (7%)	0	

SD = standard deviation.

* Any non‐vitamin K oral anticoagulant (apixaban, dabigatran, rivaroxaban) or warfarin prescribed within three months of AF diagnosis.

† Aspirin, clopidogrel, ticagrelor, or dipyridamole prescribed within three months of AF diagnosis.

The pre‐intervention incidence of newly diagnosed AF was 11 cases per 1000 eligible patients (27 cases diagnosed). During the intervention period, the incidence was 22 cases per 1000 eligible patients (screen‐detected: 17 per 1000 eligible patients; otherwise detected: 4.6 per 1000 eligible patients). The proportions of patients with stroke risk factors were similar for people diagnosed during the two periods, except that the proportion with diabetes was larger during the pre‐intervention period (9 of 27, 33% *v* 8 of 62, 13%) (Box 4).

### Stroke risk management

Guideline‐recommended prescribing of oral anticoagulation therapy for people diagnosed with AF and with CHA_2_DS_2_‐VA scores of 2 or more was similar during the pre‐intervention (20 of 24, 83%) and intervention periods (46 of 54, 85%). The management of patients at high risk of stroke was also similar for those diagnosed during the intervention with or without self‐screening (Box 4).

## Discussion

We report the first study to develop and test an AF self‐screening station fully integrated with general practice software. Self‐screening by waiting patients overcame GP time barriers, and the ECG rhythm strip and algorithm‐based evaluation were automatically imported into the patient's record, immediately available for GP review during the subsequent consultation. We found that AF self‐screening in general practices is feasible, and that it increases both screening and AF diagnosis rates. The age and stroke risk of the people who underwent self‐screening were similar to those of people diagnosed before the intervention, indicating that self‐screening targeted the appropriate population. As more than 80% of eligible patients were prescribed guideline‐recommended anticoagulation therapy, widespread adoption of AF self‐screening in general practices could reduce the incidence of stroke by detecting AF in people who might otherwise not be identified.

The AF self‐screening rate (39.8%) was almost four times as high as reported by the only other study of standard general practice AF screening in Australia (11%^6^). In one practice (which reported 24 weeks of data) the rate of AF self‐screening by eligible patients was 74%, much higher than in our earlier studies, in which GPs or nurses initiated screening during consultations (16%,^7^ 34%,^8^ 39%^13^). It is difficult to capture a large proportion of patients with GP‐ or nurse‐initiated screening because of time constraints.^7^, ^8^, ^10^, ^11^, ^13^ Self‐screening largely overcame this problem, and our model also facilitated a more uniform, practice‐wide approach to screening that did not rely on individual practitioners or practice champions.

AF self‐screening captured people at high risk of stroke, and age did not appear to be a barrier to self‐screening. These findings contrast with those of a self‐screening initiative in England that used stand‐alone waiting room AF self‐screening kiosks (Cardiocity RhythmPad) not linked with practice software.^14^ The Cardiocity initiative concluded the kiosks did not reach the target population; most who chose self‐screening were under 60 years of age, and, as the Cardiocity software was not integrated with practice software, “there was a considerable amount of variation with … the practice's ability to receive the data back in and to follow up”.^14^ Our system overcame these problems by linking self‐testing with practice software and inviting only eligible target patients; follow‐up was streamlined by automatically importing results into patient records. Older people can have difficulties using self‐screening and other self‐service health‐related technologies, reducing their uptake.^14^, ^15^, ^16^, ^17^ Our qualitative evaluation found that older people were willing to attempt self‐screening, and that the need for assistance could be reduced with further refinements to the user interface.^18^

Moving from clinician‐initiated screening to patient self‐screening is part of a broader shift to self‐service health care technologies. Self‐screening can increase efficiency, save time, and reduce costs, increasing the preventive health capacity of primary care. Self‐screening is acceptable to both GPs and patients,^18^, ^19^ but GPs have indicated that effective integration with electronic medical records and electronic decision support regarding treatment are important.^19^ Further, screening should be tailored to the health and demographic characteristics of patients and aligned with screening recommendations, such as those of the Royal Australian College of General Practitioners.^20^ Self‐screening interventions in general practice waiting rooms for depression^19^, ^21^ and hypertension^17^, ^22^ have been described, and the model could also be applied to a range of other health conditions.

The seamless integration we describe (automatic identification and notification of eligible patients, immediate transfer of screening result to the patient's medical record) is critical for a successful self‐screening program. For AF self‐screening to be offered more broadly, it is essential that the integration software be compatible with all major general practice medical record programs. A simpler user interface would also allow more patients to undertake self‐screening, making it more useful and cost‐effective. Further, the sustainability of self‐screening in general practices should be assessed, including the question of whether further support or funding is required.

### Limitations

The findings of our cross‐sectional study in a small number of NSW general practices may not be generalisable to all general practices. Bias may have been introduced by patients selecting whether to self‐screen; further, we could not determine whether all eligible patients were offered screening QR codes, how many refused them, the reasons for refusal, and how many people with QR codes did not proceed to screening. Further testing of the intervention is required to investigate participant uptake and refusal, especially by socio‐economic group, Indigenous status, and non‐English speaking background status.

As we did not collect data on further clinical investigations, we could not assess their appropriateness or calculate the cost and time burden of the intervention, particularly for following up unclassified ECGs. Reducing the proportion of unclassified results is important before implementing our approach more widely. The more recent Kardia ECG algorithm (Kardia AI V2, version 2.0.7), would reduce the proportion of unclassified ECGs from 16.8% to 1.0% (*post hoc* analysis; data not shown). The new version includes five additional diagnostic categories (sinus tachycardia, sinus bradycardia, wide QRS, atrial ectopy, ventricular ectopy), and is more tolerant of noise and artefact.^23^ It is likely that most of our unclassified results were attributable to sinus tachycardia or wide QRS (bundle branch block), which may have been pre‐existing conditions known to the patients’ GPs.

It is also important to acknowledge that anxiety is a possible harm of screening, as is inappropriate reassurance by a false negative finding.^24^, ^25^ General practitioners providing patients with timely information about the benefits and risks of screening, communicating results clearly, and completing any follow‐up as quickly as possible would have helped reduce anxiety.

Finally, COVID‐19 and related restrictions may have reduced patient participation (reduced face‐to‐face attendance; requirement to touch public screens) and caused delays in AF diagnosis, management, further investigations, and specialist referrals.

### Conclusions

We report the first investigation of an AF self‐screening station and software that identifies eligible patients and directly exports the algorithm‐based screening result to the patient's medical record. By reducing the time burden for GPs, AF self‐screening stations in waiting rooms improved AF screening and diagnosis rates. More than 80% of patients with AF and high stroke risk were treated with guideline‐recommended anticoagulation therapy. AF self‐screening in general practices could therefore reduce the number of avoidable strokes by detecting and diagnosing AF in patients who otherwise might not be identified as being at risk.

#### Data sharing statement

The de‐identified data we analysed are not publicly available, but requests for the data will be considered.

## Open access

Open access publishing facilitated by The University of Notre Dame Australia, as part of the Wiley ‐ The University of Notre Dame Australia agreement via the Council of Australian University Librarians.

## Competing interests

Katrina Giskes and Charlotte Hespe have received honoraria from Pfizer. Ben Freedman has received grants, personal fees, and non‐financial support from Bayer, BMS–Pfizer, Daiichi Sankyo, AliveCor, and Omron.

## Supporting information


Appendix S1
