# Supplementary material for: Increasing screening for atrial fibrillation in general practice: the Atrial Fibrillation Self‐Screening, Management And guideline‐Recommended Therapy (AF Self‐SMART) study
Source: Med J Aust. 2022 Dec 9;218(1):27–32. doi: 10.5694/mja2.51803 (PMC10107341; doi:10.5694/mja2.51803)
Supplement: Supplementary file 1 — Appendix S1 [file MJA2-218-27-s001.pdf]

The Medical Journal of Australia

Supporting Information

Supplementary methods and results

This appendix was part of the submitted manuscript and has been peer reviewed. It is posted as supplied by the authors.

Appendix to: Giskes K, Lowres N, Orchard J, et al. Increasing screening for atrial fibrillation in general practice: the Atrial Fibrillation Self-Screening, Management And guideline-Recommended Therapy (AF Self-SMART) study. *Med J Aust* 2023; doi: 10.5694/mja2.51803.

Supplementary methods: refinements to software during the pilot testing phase

The pilot testing phase occurred in one practice for 12 weeks, during which time the software was tested to identify any issues and bugs. Software was iteratively refined to address all issues encountered and to ensure that it accurately and consistently synced with best practice and identified the eligible patients, created valid QR codes, and accurately imported the screening ECG and diagnosis into the investigation inbox in the patient's medical record without delay.

Iterative redesign process

1. The initial screening station consisted of a table with an iPad, a KardiaMobile single lead ECG device inserted below the iPad and instructional materials printed on the table. The written instructions were incorporated into the table top itself and included text and icons representing each step of the screening process. The screening iPad was located to the right of centre, and the written instructions and instructional iPad was located on the left side of the table.
2. As reception staff were sometimes required to assist patients, the screening station was placed in a closer proximity to the reception desk, so that receptionists could give instructions without moving.
3. As it was noted that patients were having some difficult with the table-top instructions, an additional iPad with video instructions was trialled. The video featured one of the researchers who demonstrated each step of the screening process from on-boarding to completion of the screen and featured both sound and text captions. The video looped so that it was playing constantly.
4. After analysis of further staff interviews and patient observations, the following changes were made:

- The table top was altered to remove the written instructions, leaving a blank white table top only.
- The screening iPad was relocated on the table top, so as to be more central.
- The written instructions were reworked to include photographic images representing each stage of the process, to accompany the text.
- Written instructions were printed on A3 and relocated to a plastic holder so that they were vertical
- The video was changed to be user-initiated rather than looped.

Supplementary results

Table. Characteristics of participating practices and their local government areas

	Practice number						
	**1**	**2**	**3**	**4**	**5**	**6**	**Australia**
**Local government area characteristics[a]**							
Total population	43567	20842	78121	182818	213845	211632	25422789
Age (years), median	38	46	45	38	42	34	38
Born in Australia	36907 (84.7%)	17114 (82.1%)	61895 (79.2%)	114365 (62.6%)	182826 (85.5%)	94475 (44.6%)	17019815 (66.9%)
Aboriginal or Torres Strait Islander	3153 (7.2%)	1621 (7.8%)	2101 (2.7%)	2162 (1.2%)	11759 (5.5%)	3009 (1.4%)	812728 (3.2%)
Median weekly household income	$1585	$1196	$1756	$2340	$1623	$2212	$1746
IRSAD[b] (decile)	7	2	9	10	7	9	---
RRMA[c] classification	4	4	1	1	2	1	---
**Practice characteristics**							
Time in study (clinical days)	171	65	91	72	23	45	---
Total eligible patients with face-to-face GP appointment in study period	585	393	641	450	306	460	---
Number of eligible patients completed self-screening	435 (74.4%)	137 (34.9%)	303 (47.3%)	120 (26.7%)	76 (25%)	56 (12%)	---

A 2021 census data of corresponding local government areas in which practices were located.[1]

B The Index of Relative Socio-economic Advantage and Disadvantage[2] (IRSAD) summarises information about the economic and social conditions of people and households within an area, including both relative advantage and disadvantage measures. Areas in lower deciles are those with greater disadvantage and a lack of advantage. Higher deciles are indicative of areas with a lower disadvantage and greater advantage.

C RRMA (Rural, Remote and Metropolitan Area)[3] classification The RRMA classification divides Australia into 3 zones and 7 classes: metropolitan zone (RRMA 1 and 2), rural zone (RRMA 3 to 5), remote zone (RRMA 6 and 7).

References

1. Australian Bureau of Statistics. Search census data, 2021 census. Canberra: ABS, 2022. https://www.abs.gov.au/census/find-census-data/search-by-area (viewed Oct 2022).
2. Australian Bureau of Statistics. Census of Population and Housing: Socio-Economic Indexes for Areas (SEIFA), Australia, 2016. Catalogue number 2033.0.55.001 (Local Government Area, Population Distributions). Canberra: ABS, 2016. https://www.abs.gov.au/AUSSTATS/abs@.nsf/DetailsPage/2033.0.55.0012016?OpenDocument#Publications (viewed Oct 2022).
3. Australian Department of Health and Aged Care. The rural, remote, and metropolitan area (RRMA) classification. Canberra, Department of Health and Aged Care, 2021. https://www.health.gov.au/health-topics/rural-health-workforce/classifications/rrma (viewed Oct 2022).
